# Re‐Evaluating Propofol Infusion Thresholds and Delirium Risk in Mechanically Ventilated Patients: Critical Appraisal of Pharmacological Plausibility and Residual Confounding

**DOI:** 10.1002/cns.71030

**Published:** 2026-07-17

**Authors:** Shuantong Lin, Jie Zhu

**Affiliations:** ^1^ Department of Anesthesiology Eastern Bund Healthcare Shanghai China; ^2^ Department of Anesthesiology Putuo District Maternity and Child Center Shanghai China

**Keywords:** delirium, mechanical ventilation, propofol

Dear Editor,

1

We have read with great interest the recent article by Ge et al. titled “The Relationship Between the Average Infusion Rate of Propofol and the Incidence of Delirium During Invasive Mechanical Ventilation: A Retrospective Study Based on the MIMIC IV Database” published in CNS Neuroscience & Therapeutics [1]. The topic is clinically important, and the dataset is valuable. However, we have significant concerns regarding the authors' interpretation of their findings and the clinical implications they have drawn.

First, the study identifies an average infusion rate of ≥ 20 μg/kg/h sustained over 18 h as an independent risk factor for delirium (OR = 1.84, *p* < 0.001). However, this threshold appears inconsistent with established clinical practice and dosing guidelines. Current guidelines (e.g., KSCCM) [2] and randomized controlled trials [3] recommend propofol infusion rates typically ranging from 5 to 50 μg/kg/min (equivalent to 300–3000 μg/kg/h) for ICU sedation. The reported threshold of 20 μg/kg/h is approximately 100‐fold lower than the lower end of these therapeutic ranges. Critically, the authors do not clarify whether this very low rate represents effective sedation levels or subtherapeutic dosing. If the latter, the observed association with delirium may primarily reflect inadequate sedation—leading to uncontrolled pain, anxiety, or distress [4]—rather than a direct effect of propofol itself.

Second, the conclusion attributes the increased risk of delirium to propofol “accumulation” over the 18‐h period. This explanation contradicts propofol's well‐established pharmacokinetics, which are characterized by a short context‐sensitive half‐life (typically 30–60 min) and rapid hepatic and extrahepatic clearance [5]. Sustained pharmacological effects over several hours would require accumulation of the lipid emulsion carrier or its components, for which no supporting evidence is provided. A more plausible alternative explanation is that higher average infusion rates observed early during invasive mechanical ventilation (IMV) (as suggested by Figures 2a–b) may serve as markers of greater baseline illness severity or refractory agitation. These underlying conditions are themselves well‐recognized independent risk factors for the development of delirium.

Third, Table 1 demonstrates that patients with delirium had significantly higher illness severity scores (SOFA, SAPS II), lower Glasgow Coma Scale (GCS) scores, and increased mortality (*p* < 0.001). Although propensity score matching was employed, residual confounding due to illness severity likely remains. The observed association may indicate that higher infusion rates act as a surrogate marker for critical illness rather than a direct cause of delirium. This interpretation is further supported by the high odds ratio (OR = 6.08) observed in the neurological ICU (NICU) subgroup, where unit‐specific factors, such as neurological injury, may predominantly influence delirium risk.

Fourth, using an “average infusion rate” obscures temporal dynamics. A brief high‐rate infusion followed by a lower rate could misclassify a patient into a lower average exposure category, even if a significant pharmacological event occurred. This approach fails to consider the timing of delirium onset, potentially introducing misclassification bias.

In conclusion, although the study's large sample size and use of the MIMIC IV database are strengths, the conclusion implicating propofol “accumulation” at an infusion rate of 20 μg/kg/h as a significant risk factor for delirium is neither pharmacologically nor clinically substantiated. It is essential to reassess this threshold using clinically relevant infusion rates and to conduct further analyses that more effectively address confounding factors such as illness severity and treatment indications.

## Conflicts of Interest

The authors declare no conflicts of interest.

## Linked Article

This article is linked to Gei et al. papers. To view these articles, visit https://doi.org/10.1111/cns.70273.

## Data Availability

Data sharing not applicable to this article as no datasets were generated or analysed during the current study.
